# Antitumor Activity of 1-Triphenylgermyl-4-Propiono-substituted Semicarbazides, Thiosemicarbazides and Their Heterocyclic Derivatives

**DOI:** 10.1155/MBD.1996.241

**Published:** 1996

**Authors:** Fengfu Li, Zhongbiao Zhang, Huan Gao

**Affiliations:** 1 Institute of Elemento-Organic Chemistry Nankai University Tianjin 300071 China; 2 Institute of Polymer Chemistry Nankai University Tianjin 300071 China


					ANTITUMOR ACTIVITY OF 1-TRIPHENYLGERMYL-4-PROPIONO-
SUBSTITUTED SEMICARBAZlDES, THIOSEMICARBAZlDES
AND THEIR HETEROCYCLIC DERIVATIVES
Fengfu Li1, Zhongbiao Zhang and Huan Gap.2
Institute of Elemento-Organic Chemistry 2 Institute of Polymer Chemistry
Nankai University, Tianjin, 300071, P. R. China
ABSTRACT
Five organogermanium compounds with the formulae Ph3GeCHR' CH.CONHNHC(X)NHR" and
N--NH
Ph3GeCHR' CH.C C X
N
at!
( R' H, Ph; R" Ph, p-CH3-Ph X S, O) were found to possess inhibitory effects on gastric
carcinoma MGC-803 in vitro.
Key words: germanium, organogermanium, antitumor activity
It has been reported that trialkylgermylpropanoic acids and their derivatives showed antibacterial
activity[1
and the selectively inhibitory action on the decomposition of enzymes[2. However, no
antitumor properties of these compounds have been known in the literature. In our previous work;,
we have reported the syntheses of some 1-triphenylgermyl-4-propiono-substituted semicarbazides,
thiosemicarbazides (1) and their heterocyclic derivatives (2). In the present paper, we report the
antitumor activity of these compounds.
Table
Compounds
Effects of 1 and 2 on gastric carcinoma MGC-803
Inhibition rate (
lppm lOppm lOOppm
la 38.4 32.8 18.4
lb 20.0 28.8 60.8
lc 8.0 21.6 28.0
2a 28.0 40.0 48.0
2b O. O0 4.80 15.2
Inhibition rate reported in this paper was tested according to reference 4.
Dimethyl sulfoxide was used as solvent, the same in Tables 2 and 3.
Table 2 Effects of 1 and 2 on gastric carcinoma BGC-823
Compounds Inhibition rate ( o)
lppm lOppm lOOppm
la -6.82 -12.5 -1.55
lb -4.55 -2.27 68.18
lc -14.77 -9.09 19.32
2a -9.09 -4.45 9.09
2b -34.15 -15.85 -6.10
241
Vol. 3, No. 5, 1996 AntitumorActivity of1-Triphenylgermyl-4-Propiono
Table 3 Effects of 1 and 2 on nasopharyneal darcinoma KB
Compounds Inhibition rate ( %
lppm lOppm lOOppm
la -11.63 -8.53 -3.10
lb -7.75 20.93 56.59
lc -1.55 4.65 25.58
2a -13.18 7.75 18.60
2b -6.20 -9.30 -3.80
Ph3GeCHR' CHzC(O)NHNHC(X)NHR"
N--NH
Ph3GeCHR' CHzC C X
N
a
la
lb
lc
1
R' --H, X--S ,R" p-CH-Ph
R' --Ph, X--S ,R" p-CH-Ph
R --Ph,X--O,R" =Ph
2a
2
R --H ,X--S ,R" =p-CH-Ph
R' --Ph,X--O ,R" =Ph
As shown in Table 1, to some extent, compounds 1 and 2 are all effective against gastric
carcinoma MGC-803 under the experimental conditions. However, no inhibitory effects were found
against gastric carcinoma BGC-823 or nasopharyneal darcinoma KB under the same conditions as
above (see Tables 2 and 3).
Acknowledgement. The authors are grateful to the State Key Laboratory of Elemento-Organic
Chemistry for financial support of this project, also to Beijing Medical University for their testing of
antitumor activity.
REFERENCES
I. Kakimoto, N; Yashihara, T Akiba, M. and Takada, T. Japanese Patent, 6200093: CA,106,
196613b.
2. Kakimoto, N Katayama, Y; Mori, M. and Hasato, Y. ibid, 6200092 :CA, 106,196614c.
3. Chert, R. and Li, F., Appl. Organomet. Chem., 1995, 9, 277.
4. Denizot, F. and Lang, R. Journal of Immulogical Method,1986, 89, 271.
Received- May 7, 1996 Accepted" June 7, 1996
Received in revised camera-ready format: October 3, 1996
242

				

